# Unexpected endemism in the *Daphnia longispina* complex (Crustacea: Cladocera) in Southern Siberia

**DOI:** 10.1371/journal.pone.0221527

**Published:** 2019-09-03

**Authors:** Elena I. Zuykova, Nickolai A. Bochkarev, Derek J. Taylor, Alexey A. Kotov

**Affiliations:** 1 Laboratory for ecology of vertebrate communities, Institute of Systematics and Ecology of Animals of Siberian Branch of the Russian Academy of Sciences, Novosibirsk, Russia; 2 Department of Biological Sciences, The State University of New York at Buffalo, Buffalo, New York, United States of America; 3 Laboratory of aquatic ecology and invasions, A.N. Severtsov Institute of Ecology and Evolution of Russian Academy of Sciences, Moscow, Russia; University of Georgia, UNITED STATES

## Abstract

The biological significance of regional cladoceran morphotypes in the montane regions of the central Palearctic remains poorly understood. In the Holarctic *Daphnia longispina* complex (Cladocera: Daphniidae), several variants, lineages and species have been proposed as endemic for Southern Siberia. *Daphnia turbinata* Sars, for example, named after its unusual head shape, is known only from Southern Siberia. Here we sequence DNA of *Daphnia* from three mitochondrial genes (12S rRNA, 16S rRNA, and NADH dehydrogenase subunit 2, *ND2*) from 57 localities in Russia and Mongolia (the majority being from Southern Siberia) and place them in evolutionary context with existing data. Our aim was to examine regional endemism of the *Daphnia longispina* complex in Southern Siberian; to improve the phylogenetic understanding with improved taxonomic and regional sampling, and to better understand the influence of Pleistocene glaciation on the biogeography of these lineages. At least three lineages showed genetic evidence for endemism in Southern Siberia. There was strong support for *D*. *turbinata* as a sister lineage to to *D*. *longispina*/*D*. *dentifera*. Another endemic, Siberian *D*. cf. *longispina*, is a sister group to the *longispina* group in general. Within *D*. *longispina* s. str. there was an endemic Siberian clade with a western range boundary near the Yenisei River Basin. Gene flow estimates among populations (based on *F*_ST_ values) were very low for clades of *D*. *longispina* on a regional (the original *12S* dataset), and on a pan-Eurasian (the extended *12S* dataset) scale. Negative values of Fu’s *F*_S_ and Tajima’s *D* tests prevailed for the species examined with significant values found for two *D*. *longispina* clades, *D*. *dentifera*, *D*. *galeata* and *D*. *cristata*. Our results support the notion that Southern Siberia is an important biogeographic region for cladocerans as it contained unexpected diversity of endemics (such as *D*. *turbinata*, *D*. cf. *longispina* and lineages of *D*. *umbra* and *D*. *longsipina* s.str.) and from being the geographic meeting place of expanding postglacial lineages from eastern and western refugia.

## Introduction

Water fleas (Crustacea: Cladocera) are model organisms for evolutionary biologists, hydrobiologists and biogeographers. In the last decade of the 20th century and first decade of the 21st century, molecular genetic methods were intensively used for cladocerans [1, 2, 3, 4, 5, 6, 7]. This application of molecular methods led to rapid progress in taxonomy and evolutionary biology. For cladocerans, an integrated approach has been ideal—combined morphological and molecular methods helped to define boundaries among congeneric taxa and revealed undescribed species [8, 9, 10, 11, 12, 13, 14]. But the identification of "problematic" (e.g. hybrid) specimens, populations and species in the *D*. *longispina* group remained difficult [15]–moreover cases of mito-nuclear discordance between provisionary "taxa" were proposed [16]. Still, molecular analyses alone have been important within this group when morphological redescriptions and diagnostic characters were lacking [17, 18]. For example, molecular studies (and in some cases paleolimnological records) supported rapid postglacial evolution of the “defensive” morphotypes in cladocerans [6, 19, 20]. As with fish, the significance of postglacial morphotypes for speciation and taxonomy remains actively researched. Moreover, older pre-glacial “defensive” (i.e., “helmet” and carapace shape variants) morphotypes of cladocerans may yet exist in the relatively unexamined fauna of central Palearctic montane regions.

Molecular studies revealed numerous cases of cryptic species within different animal groups [21, 22, 23], including different families of water fleas [12, 24, 25, 26, 27]. For the *Daphnia longispina* group, highly divergent mitochondrial lineages have been detected in different geographic regions of Eurasia [7, 28; 29, 30]. Often, existing morphological keys are inadequate to recognize divergent lineages related to *D*. *longispina* (e.g., "*D*. *curvirostris*" in Japan [31] and *D*. *lacustris* in Europe [32]). It is obvious that the application of recent keybooks do not allow us to resolve the exact taxonomic status of problematic populations—additional morphological investigations are necessary to find their diagnostic characters. Moreover, molecular data suggest a hybrid status of some divergent lineages of *D*. *longispina* [16], and this could reflect some ancient events in the evolutionary history of the group, as well as its ancient polymorphism. It is possible that lineages bearing ancient mitochondrial DNA survived in refugia during the Pleistocene glacial cycles. Phylogeographic studies based on different taxa in different geographic regions strongly suggested the existence of such refugia [33, 34, 35, 36, 37, 38, 39]. Some refugia were located in montane regions, which are now well-known sources of endemic cladocerans [40, 41, 42].

Still, little is known of cladoceran biogeography in the montane regions beyond Europe. [36, 42]. Western and Eastern Siberia are among the most vast and understudied regions in cladoceran biogeography [42]. Only a few trans-Palaearctic phylogeographic studies exist. These studies suggested a strong longitudinal differentiation of the fauna within the Palearctic [26, 27, 43, 44].

Our previous studies of the genetic structure of the *D*. *longispina* complex in Siberian populations revealed unexpected taxa: *D*. *umbra*, *D*. *dentifera*, a presumably new taxon from Western Siberia and several divergent mitochondrial lineages of *D*. cf. *longispina* [45, 46, 47, 48, 49]. These initial studies indicated the potential for endemic species and haplotypic structure in the *D*. *longispina* complex of mountain and pre-mountain water bodies of Southern Siberia. Even before molecular studies, Sars [50, 51] pointed to the existence of some endemic taxa in this region, including *Daphnia longispina* var. *turbinata*. The proposed variant with an unusual head shape was described from the basin of Teletskoe Lake in the Altai Mountains and then recorded from some water bodies of Mongolia and Baikal region [52, 53]. Glagolev [54] concluded from morphology that *Daphnia turbinata* Sars is a valid taxon from the *D*. *longispina* complex. Still, the phylogenetic position of this taxon is unclear and no available genetic data exists for *D*. *turbinata*.

Here we aimed: (1) to evaluate the level of endemism for the *D*. *longispina* complex in Southern Siberia based on the sequences of three mitochondrial genes (*12S*, *16S* and *ND2*); (2) to analyse the geographic distribution of common and rare taxa of the *D*. *longispina* complex and their haplotypes in the water bodies within this region; (3) to study in detail the genetic structure of *D*. *longispina*, the most common taxon in this region.

## Material and methods

### Ethics statement

The study did not involve any endangered or protected species. Field collection in Russia was carried out by our team or by colleagues as part of a governmental project "Ecology and biodiversity of aquatic ecosystems and invasions of alien species" (№ 0109-2014-0008 for 2015–2017) and the Federal Fundamental Scientific Research Program for 2013–2020 № VI.51.1.9. (AAAA-A16-116121410119-4), with governmental permission to collect samples from public property. Sampling in the natural reserves of Russia (Azas Federal Natural Reserve and former Belozersky Zakaznik) was conducted with special permissions of their Administration. Mongolian samples were collected by the Joint Russian-Mongolian Complex Biological Expedition with permission of the Ministry of Nature, Environment and Tourism of Mongolia.

### Sampling

Zooplankton samples were collected by the Juday-type (125 μm mesh size) and Apstein-type (250 μm mesh size) plankton nets during summer season of 2004–2017, fixed in 96% ethanol immediately after collecting, and then stored at –20°C. Prior to DNA extraction, each *Daphnia* specimen was photographed in lateral view using an Altami microscope (Altami, Russia, under 4× and 10×) for documentation of its body and head shape. As possible, each specimen was identified to species level according to existing keys [55].

### DNA extraction and sequence analysis

**Fig 1:**
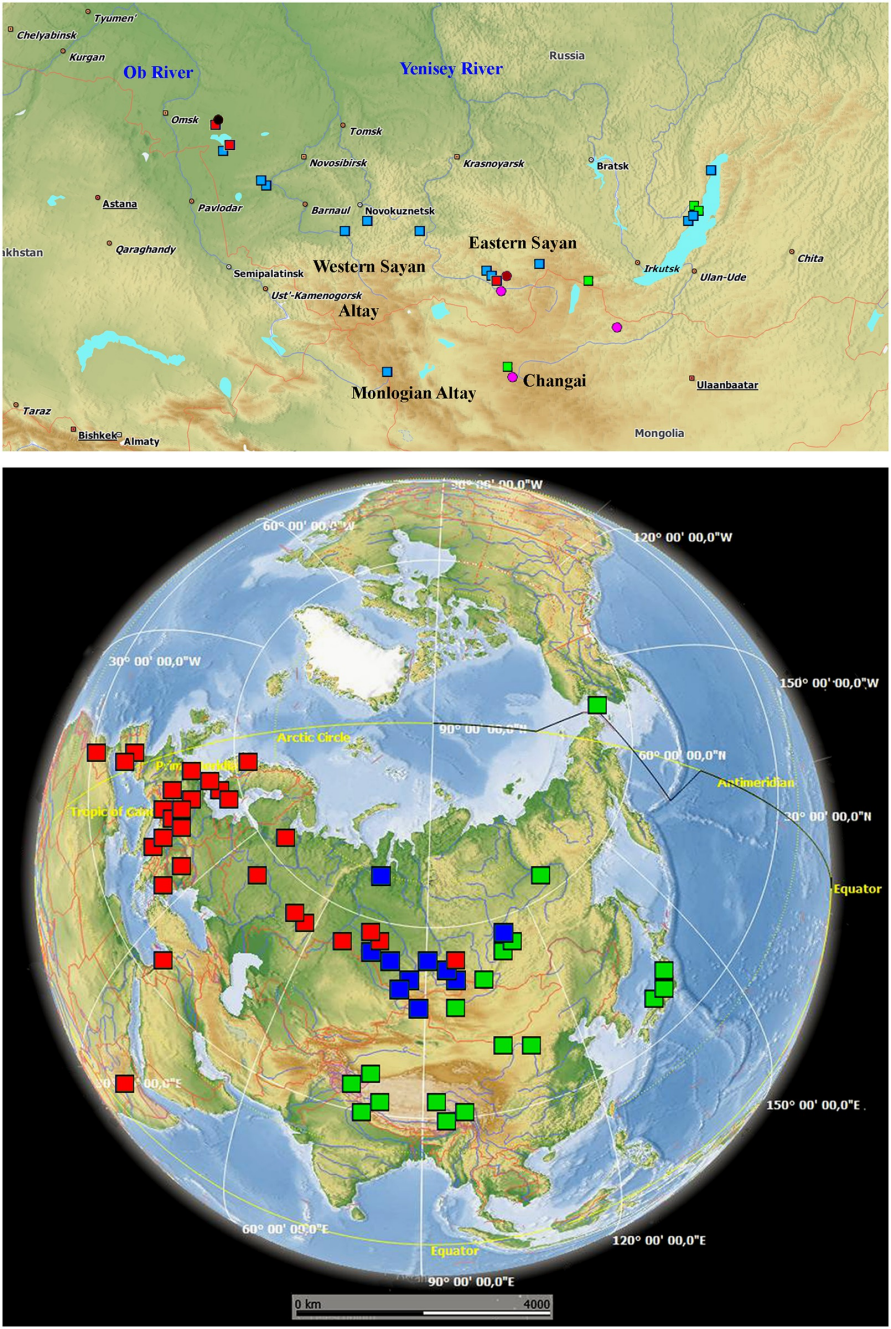
Distribution of the taxa and phylogroups of *Daphnia longispina* complex in Eurasia. Upper panel—a map of Southern Siberia and Mongolia; lower panel—global map. Shapes of different colors correspond to different species, namely: red squares, *D*. *longispina* clade B; blue squares, *D*. *longispina* clade A; green squares, *D*. *dentifera*; black circle, *D*. cf. *longispina*; pink circles, *D*. *turbinata*; brown circle, *D*. *umbra*. The base map for lower panel was obtained from the open domain plain map available at https://marble.kde.org/.

Original sequences were obtained here for specimens from 57 localities of Russia and Mongolia (Fig 1, S1 Table). Most these water bodies were located in the Southern Siberia in basins of large Siberian rivers: Yenisei (with its largest affluent, the Angara, starting from Lake Baikal) and Ob (with its largest affluent, the Irtysh), but some additional samples from Yakutia (Lena basin), Ural Mountains, Kamchatka Peninsula and European Russia are added.

Total genomic DNA was extracted using a 5% suspension of Chelex 100 resin (Bio-Rad, USA) from single ethanol-preserved *Daphnia* specimen. One to ten individuals per population were selected for mitochondrial DNA analysis. One to three mitochondrial markers were amplified for each specimen, namely: two ribosomal RNA genes: a 528–529 bp fragment of the *12S* and a 476–477 bp fragment of the *16S* genes, and 718 bp protein-coding fragment of the NADH dehydrogenase subunit 2 (*ND2*) gene. The PCR conditions and protocols were as specified previously [45, 48]. The PCR products were separated on 0.9–1% agarose (Low EEO Standart agarose, BIOZYM, Russia) in the presence of ethidium bromide and photographed under UV light. The amplified products were purified using a kit from BIOSILICA (Novosibirsk, Russia) and the samples were sequenced in both the forward and reverse direction at the company “Syntol” (Moscow, Russia, www.syntol.ru). The newly obtained nucleotide sequences were deposited into the GenBank under the following accession numbers: MN251883-MN251898, MK930508-MK930512, MK930467-MK930484, MK951805-MK951810 for the *12S* gene; MK930485-MK930487, MK930489; MK930490; MK930492; MK930493 for the *16S* gene; and MK930499-MK930505 for the *ND2* gene (see accession numbers see S1 Table).

### Mitochondrial datasets

The sequences for the species of the *D*. *longispina* complex were grouped into three mitochondrial datasets. The first dataset was composed of 477 original nucleotide sequences and sequences obtained from the GenBank database (S2 Table). Hereafter, this dataset was designated as “extended *12S* dataset”. The second dataset was named “original *12S* dataset” and comprised of 150 original sequences (of differing ages). The third analyzed dataset was named as “concatenated *12S*+*16S*+*ND2* dataset” and was composed of 49 original nucleotide sequences (S1 Table).

### Phylogenetic analyses

The nucleotide sequences were automatically aligned using the ClustalW algorithm [56] and then manually edited using BioEdit v.7.0 [57]. Then datasets were tested for redundancy and saturation and were collapsed into haplotypes using METAPIGA v.3.01 [58], when necessary.

The best-fitting models of nucleotide substitution for both *12S* rRNA gene datasets were selected in jModelTest v. 2.1.7 based on the likelihood scores for 88 different models and under the Akaike Information Criterion (AIC) and Bayesian Information Criterion (BIC) [59, 60]. The best models were General Time Reversible with invariant sites and gamma distribution (GTR+I+G, α = 0.38 [61]) for the extended *12S* dataset and Tamura-Nei with gamma distribution (TrN+G, α = 0.25 [62]) for the original *12S* alignment. The phylogenetic trees based on both *12S* datasets were reconstructed in MEGA v. 7.0 using the maximum likelihood (ML) algorithm with pairwise deletion of the gaps and missing sites [63]. One thousand bootstrap replicates were run to assess the statistical support for the tree nodes [64]. Bayesian analysis was performed with MrBayes v.3.2 [65] under the GTR+I+G (extended *12S* dataset) and GTR+G (original dataset) models. Two simultaneous runs with four Markov chains each were run for 1×10^6^ (original dataset) and 10×10^6^ generations (extended dataset) and sampled every 500 generations. Convergence of runs was assessed by examination of the average standard deviation of split frequencies and the potential scale reduction factor. In addition, stationarity was confirmed by examining posterior probability, log likelihood, and all model parameters by the effective sample sizes (ESS > 200) and trace plots of MCMC output in the program Tracer v.1.7 [66, 67].

For the concatenated dataset we determined the best-fit models of nucleotide substitution and the optimal partitioning scheme using PartitionFinder v.2 [68] and IQ-TREE v.1.5.4 [69, 70] under the AIC and BIC. The partition schemes selected by IQ-TREE were subsequently used in the ML search with the same software, using 1000 ultrafast bootstrap replicates [71]. The following partition schemes for ML were selected by the corrected AICc: for *12S* rDNA, *16S* rDNA and *ND2* codon position 3 (HKY+F+R2); *ND2* codon position 1 (HKY+F+I); *ND2* codon position 2 (TN+F). The tree support was accessed with the rapid-bootstrapping algorithm using 1000 non-parametric bootstrap replicates. Bayesian analysis for the concatenated dataset was performed with MrBayes v.3.2 under the following partition schemes: *12S* rDNA (GTR+G); *16S* rDNA (GTR+I+G); *ND2* codon position 1 (GTR+I); *ND2* codon position 2 (GTR); *ND2* codon position 3 (GTR+G). Two simultaneous runs with four Markov chains each were run for 1×10^6^ generations and sampled every 500 generations. The first 25% of generations were discarded as burn-in. Convergence of runs was assessed by examination of the average standard deviation of split frequencies and the potential scale reduction factor. The stationarity was confirmed as indicated above by the effective sample sizes (ESS > 500) and trace plots in the Tracer v.1.7. The phylogenetic trees resulting in ML and BI analyses were visualised and edited using FigTree v.1.4.3 [72]. The sequences of *D*. cf. *longispina* and *D*. *umbra* were used as outgroup rooting of the original and concatenated phylogenetic trees; and *D*. *cristata* was used for the extended *12S* phylogeny.

A split network as an alternative method of analysis was performed using NeighborNet model with "equal angle" algorithm and uncorrected *p*-distances in the SplitsTree v.4.10 [73, 74] based on the original *12S* nucleotide sequences for all studied species of the *D*. *longispina* complex collapsed into haplotypes. A split network robustness was tested using 1000 bootstrap replicates. The haplotype networks were constructed by the median-joining method (MJ) [75] using Network v.5.0 (available on www.fluxus-engineering.com) based on the original *12S* sequences for the *D*. *longispina* clades.

### Genetic diversity, population structure and neutrality tests

The mitochondrial DNA polymorphism for the studied species and two clades of *D*. *longispina* was estimated separately for both *12S* datasets and the concatenated alignment. The following parameters were calculated using DnaSP v.5.10 [76]: the number of haplotypes (*h*), number of segregating sites (*S*), haplotype diversity (*H*_d_), and nucleotide diversity (π).

A hierarchical analysis of molecular variance (AMOVA) for the *D*. *longispina* and *D*. *dentifera* populations was conducted using Arlequin v.3.5.2.2 [77]. Three AMOVAs were carried out to examine patterns of genetic differentiation into (1) the “among geographical groups”, “among populations within groups” and “within populations” components; (2) the “among populations” and “within populations” components; (3) the “among clades”, “among populations within clades” and “within populations” components. For this all *D*. *longispina* populations were grouped into eight geographical groups (S3 Table); and *D*. *dentifera* was grouped into three groups (S4 Table). This analysis was performed for the *D*. *longispina* populations and clades based on extended and concatenated datasets; and for *D*. *dentifera* populations the analysis was based on the extended dataset only. The significance of Φ-statistic parameters was assessed by permutation tests with 10000 replicates as implemented in Arlequin v. 3.5.2.2.

An average evolutionary divergence over original *12S* sequence pairs within and between clades and species of the *D*. *longispina* complex using uncorrected *p*-distances was estimated in MEGA v.7.0. To assess genetic distances among populations, pairwise *F*_ST_ values were calculated using the extended dataset with Arlequin v.3.5.2 and associated probability values were calculated using 10 000 permutations. Then, the pairwise *F*_ST_ comparisons were plotted. The neutrality tests of Fu’s *F*_S_ [78] and Tajima’s *D* [79] were calculated for the species and clades of the *D*. *longispina* complex with DnaSP v.5.10 to investigate the historical population demographics and testing whether the sequences conformed to the expectations of neutrality. The significance of these tests was proved using the coalescent simulation with 1000 permutations.

## Results

### Phylogeny and haplotype distribution

#### The extended *12S* rRNA gene dataset

According to BI and ML analyses, all *12S* sequences of the *D*. *longispina* complex are subdivided into seven specific clusters; tree topologies were identical in both analyses (S1 Fig). The eighth cluster is composed of *Daphnia cristata* sequences and was used as an outgroup. Most parts of the clusters were monophyletic except for *D*. cf. *longispina* and *Daphnia* sp. from Berse, which have unclear positions in the overall phylogeny of the group. There are multiplied lineages (subclades) within each specific cluster with branch support up to 100%. Within the *D*. *longispina* sequences, a distinct clade A is clearly distinguished; it is formed by the haplotypes from remote mountain water bodies of Siberia, while a clade B is widely distributed in Eurasia and includes the type locality of *D*. *longispina*, Denmark (see arrow in S1 Fig). The haplotypes of *D*. *dentifera* from the water bodies of Yakutia and the Baikal region pooled into one subclade (91%)–the Yakutia sequences formed a distinct group within *D*. *dentifera* (100%). *D*. *turbinata* is a sister group to the *D*. *longispina*—*D*. *dentifera*—*D*. *galeata*—*D*. *cucullata* cluster. The *D*. *umbra* and *D*. *lacustris* haplotypes form a distinct monophyletic clade (91%), and several divergent subclades are found within the *D*. *umbra* cluster.

#### The original *12S* dataset

**Fig 2:**
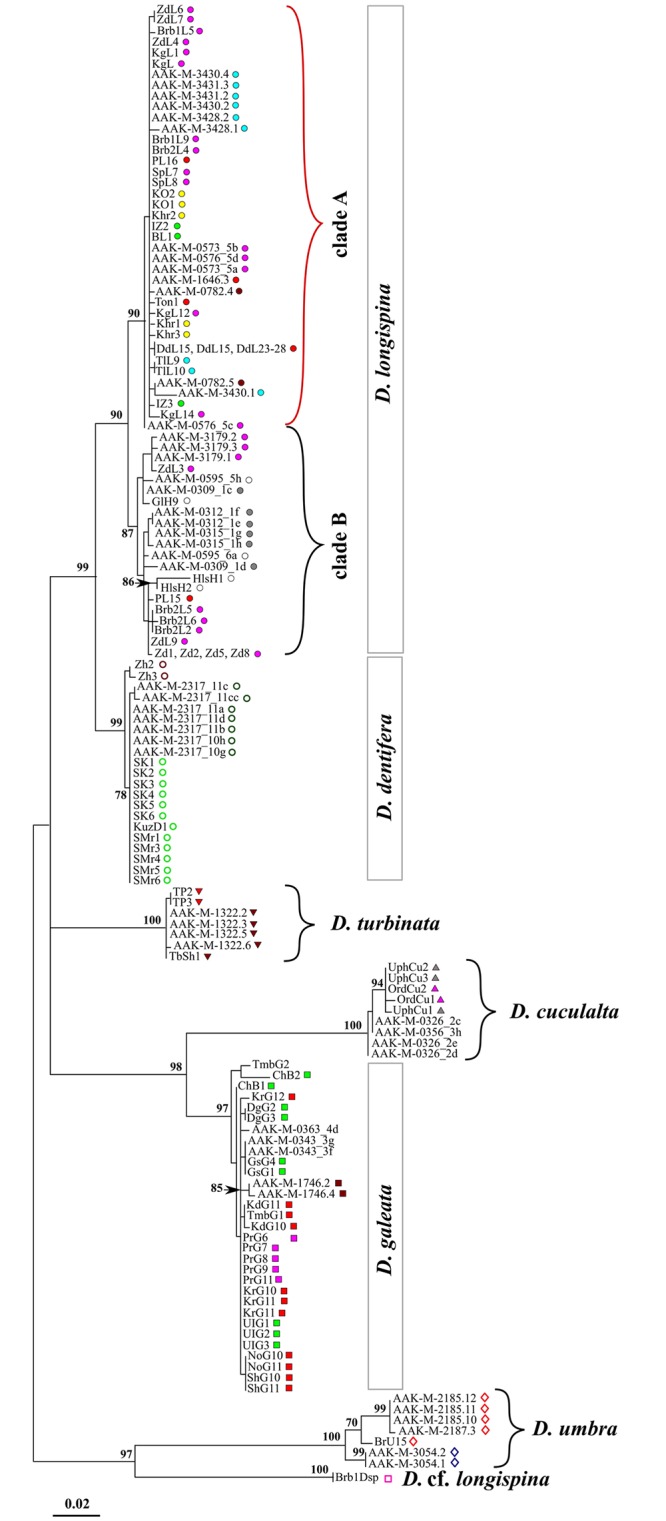
Maximum likelihood phylogenetic tree for species of the *D*. *longispina* complex based on the original *12S* dataset. ML bootstrap values above 70% are indicated for each significant node. Scale is given in expected substitutions per site. Colored geometric symbols are the same as in Fig 1 and S1 Fig.

The reconstruction of phylogenetic relationships within the *D. longispina* complex based on the original *12S* sequences of the mtDNA revealed seven strongly supported clusters, which reflect the nominative species (Fig 2). However, the analysis of the original *12S* sequences, failed to resolve the position of *D. turbinata* with respect to other species of this complex. Moreover, a discordance between the ML and BI phylogenetic trees was found (S2 Fig). The closely related species *D. longispina*—*D. dentifera*, *D. galeata*—*D. cucullata*, as well as *D. umbra—D*. cf. *longispina* comprised some separated clusters with a significant support. The cluster joined of the last two above-mentioned species can be considered as outgroup in relation to other species of the complex. According to the BI analysis, the *D. umbra—D.* cf. *longispina* sequences formed the pooled sister lineage to the *D. turbinata* sequences (S2 Fig). All *D. longispina* sequences could be clearly separated into two large clades; at the same time the haplotypes within clade B were subdivided into a number of subclades with a strong support values (Fig 2, S2 Fig). The haplotypes from Mongolia were clearly divided into distinct groups within the *D. galeata* cluster. The *D. umbra* haplotypes from different geographical regions also forming divergent subclades.

**Fig 3:**
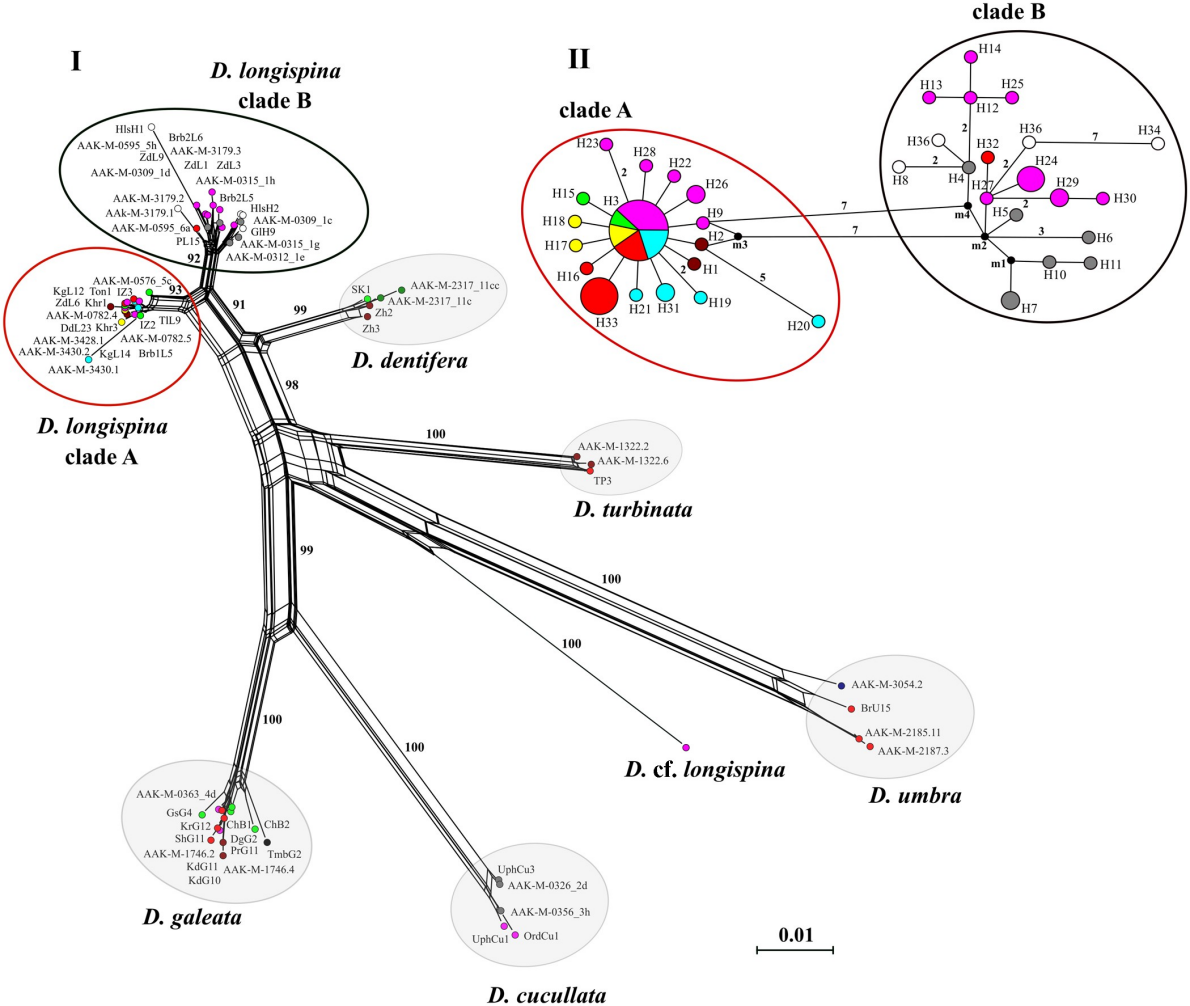
**Network phylogenies based on the original *12S* dataset: (I) Split tree for species of the *D*. *longispina* complex with uncorrected *p*-distances.** Bootstrap support is shown for each split; scale is expected substitutions per site. **(II) Median-joining network for haplotypes of the *D*. *longispina* clades.** Each circle of MJ network is proportional to relative haplotype frequencies (scale is shown in the upper right corner). The numbers of mutations are labeled for each branch (if not 1). Colors are the same as in Fig 1 and S1 Fig; ID for haplotypes see S1.

In general, the structure of the split network based on the original *12S* dataset of the *D*. *longispina* complex coincided with the ML-tree topology (Fig 3I). It is interesting, that the position of *D*. *turbinata* was even more close to the *D*. *longispina*–*D*. *dentifera* cluster in this analysis, than in the ML and BI phylogenies. The bootstrap supports were high (from 92 to 100%). The median-joining network for the original *12S* haplotype of *D*. *longispina* unambiguously confirmed the existence of two clades (Fig 3II). Clade A was characterized by a star-shaped structure with the central haplotype H_3 occurring in the populations from different regions of Siberia. Nevertheless, the bulk of haplotypes of this clade was found in the mountain water bodies of the Altai-Sayan highland and the basin of Lake Baikal (Fig 3II). Clade B was composed mainly of the *D*. *longispina* haplotypes from the water bodies of the Ob-Irtysh basin. The exception was a single haplotype from an unnamed pond situated at the Todzha Depression (the Yenisei River basin). Several haplotypes from the Urals and Eastern and Central Europe also belonged to clade B. There are seven substitutions and three hypothetical haplotypes between the clades A and B.

#### The concatenated dataset

**Fig 4:**
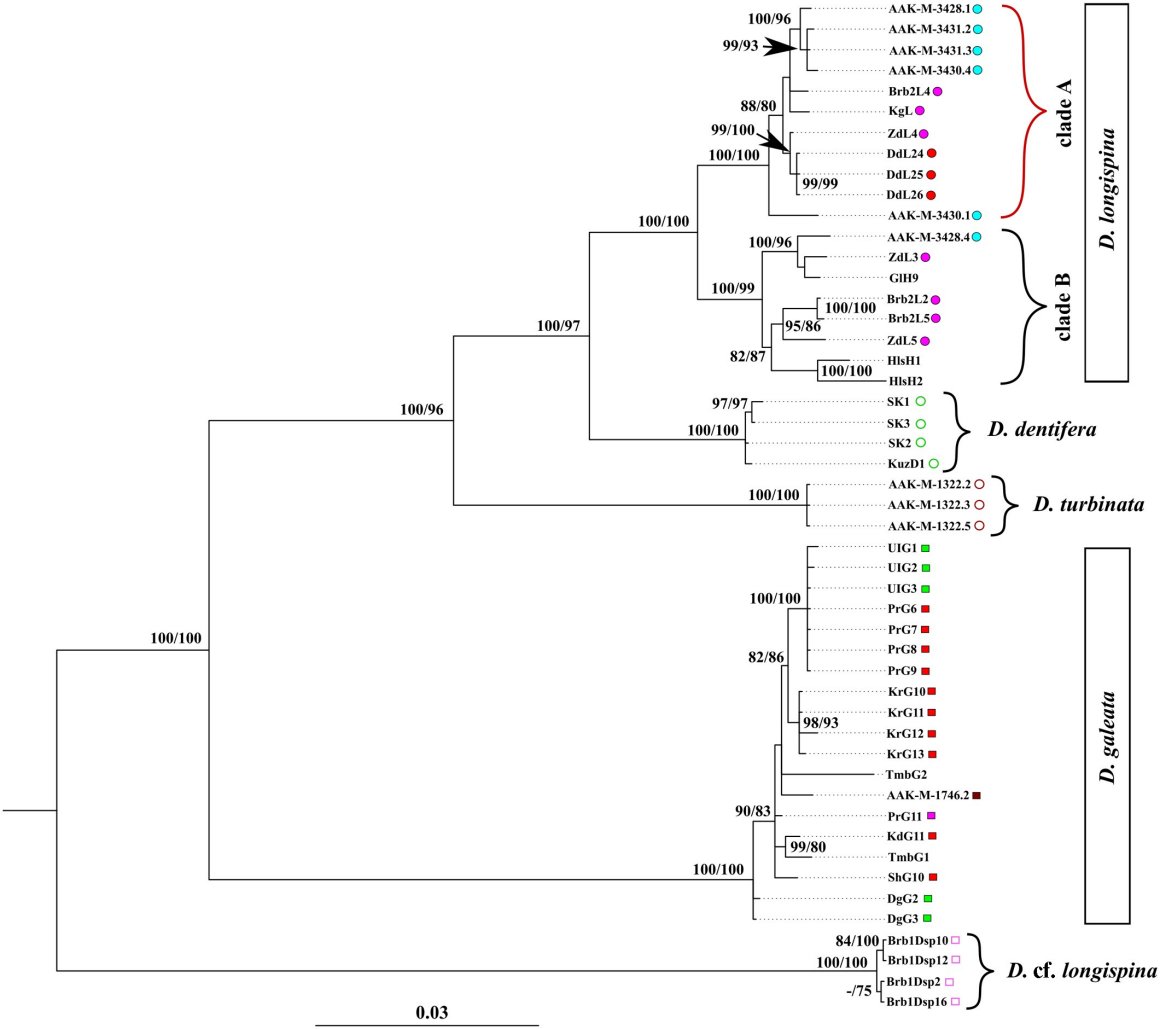
Bayesian phylogenetic consensus tree for *D*. *longispina* based on the concatenated (*12S* + *16S* + *ND2*) dataset. Bayesian posterior probabilities BI and bootstrap values from ML analysis above 75% expressed as a percentage are indicated for each significant node. The scale is given in expected substitutions per site. Colored geometric symbols are the same as in Fig 1.

According to the BI and ML analyses, all concatenated *12S*+*16S*+*ND2* sequences of the *D*. *longispina* complex were subdivided into five clusters also corresponding to the aforementioned species (Fig 4). In general, clusters corresponded to the clusters of our *12S*-based phylogenies. The exceptions were the *D*. *cucullata* and *D*. *umbra* clusters, which were not presented in the current analysis. The concatenated-tree topology revealed strong support for the monophyly of the *D*. *longispina*–*D*. *dentifera*–*D*. *turbinata* clade (100/96). This result was concordant with that obtained for the extended *12S* tree. The monophyly of this clade and *D*. *galeata* was also supported with this analysis.

Additionally, the analysis of the concatenated dataset also supported the existence of two major *D*. *longispina* clades. The divergence between them was even deeper than that in the *12S*-phylogenies. Each specific cluster as well as the *D*. *longispina* clades contained one or more divergent mitochondrial haplogroups with high branch supports (Fig 4). Among such haplogroups, clade A includes specimens from the lakes of the Altai Mountains (upstream of the Ob River basin), and another clade includes specimens from Lake Dodot (upstream of the Yenisei River basin). Clade B was formed by three groups of haplotypes; there was an additional inner divergent lineage in one of these groups encompassing the sequences from the temporary water bodies of the Lake Chany basin. The second haplogroup consisted of the sequences from three geographically distant *D*. *longispina* populations, namely from the Altai Mountains, the Lake Chany basin and Lake Glubokoe (located in European Russia). And the third group united the "*D*. *hyalina"* haplotypes from Lake Hallstättersee (Austria). As for *D*. *dentifera*, there was a divergent lineage uniting sequences from the population from Lake Srednee Kedrovoye (the Lake Baikal basin).

The concatenated *12S*+*16S*+*ND2* sequences of *D*. *galeata* were subdivided into three divergent haplogroups. The first group joined the haplotypes from the Ust-Ilimsk Reservoir (the Angara River, the Lake Baikal basin) and Lake Bolshoye (the Ob River basin). The second haplogroup united the haplotypes from Lake Karakul. The third group unitesd the haplotypes from the geographically distant *D*. *galeata* populations, namely from Lake Kadysh (the Todzha Depression, the Yenisei River basin) and from an unnamed pond near Vladivostok city (the Far East of Russia). The concatenated sequences of *D*. cf. *longispina* were also divided into two groups (Fig 4).

### Genetic polymorphism and population structure

In toto, the level of genetic polymorphism of the concatenated fragment of the mtDNA is higher than that of *12S* gene in all studied species of the *D*. *longispina* complex (Table 1, S5 Table). This is especially obvious when analyzing the level of haplotype diversity (*H*_d_) in *D*. *dentifera*. Clade B of *D*. *longispina*, *D*. *dentifera* and *D*. *galeata* are characterized by higher nucleotide diversity (π) as compared to other species. *D*. *longispina*, *D*. *galeata* and *D*. *umbra* have a large number of haplotypes (*h*) and polymorphic sites (*S*) (Table 1, S5 Table). The highest *H*_d_ and π values are found in *D*. *umbra*.

**Table 1:** Polymorphism of the mtDNA based on the original *12S* and concatenated *12S*+*16S*+*ND2* datasets for the studied *Daphnia* species and *D*. *longispina* clades. Abbreviations: con, concatenated fragment (*12S*+*16S*+*ND2*); *n*, number of sequenced *Daphnia* individuals; *S*, number of polymorphic sites; *h*, number of haplotypes; *H*_d_, haplotype diversity; π, nucleotide diversity; st.d., standard deviation.

Species	*n*		*h*		*S*		*H*_d_ ± st.d.		π ± st.d.	
	*12S*	con	*12S*	con	*12S*	con	*12S*	con	*12S*	con
*D*. *longispina* clade A	46	11	17	9	22	32	0.768±0.059	0.945±0.066	0.0024±0.0005	0.0045±0.0011
*D*. *longispina* clade B	24	8	19	8	28	73	0.971±0.024	1.000±0.063	0.0088±0.0011	0.0149±0.063
*D*. *dentifera*	21	4	5	4	6	4	0.352±0.131	1.000±0.177	0.0015±0.0006	0.0013±0.0004
*D*. *turbinata*	7	3	3	1	2	0	0.667±0.160	0	0.0014±0.0004	0
*D*. *cucullata*	9	‒	5	‒	7	‒	0.861±0.087	‒	0.0049±0.0008	‒
*D*. *galeata*	28	19	12	14	22	64	0.831±0.063	0.936±0.047	0.0046±0.0012	0.0060±0.0011
*D*. *umbra*	7	‒	4	‒	17	‒	0.810±0.130	‒	0.0150±0.0033	‒
*D*. cf. *longispina*	5	4	1	2	0	1	0	0.667±0.204	0	0.0004±0.0001

A hierarchical AMOVA supported a high level of the genetic subdivision of *D*. *longispina* and *D*. *dentifera* as with the phylogenetic reconstruction. In general, there was a strong within-population structuring. Considering the hierarchical level “geographical areas” and the unstructured dataset, AMOVA analysis showed a significantly high molecular variance for the “within populations” component, 55.22% for *D*. *longispina* (Table 2) and 77.93% for *D*. *dentifera* (Table 3). When we considered the “*D*. *longispina* clades” level, the AMOVA assigned the main portion of molecular variance to the “among clades” level (71.77–73.39%) whereas populations within clades showed low variation (5.68%). All Φ-statistics were highly significant (*P* < 0.001 or *P* < 0.05), except for the“within populations” component at the hierarchical “*D*. *longispina* clades” level and the unstructured dataset of *D*. *dentifera* (Tables 2 and 3).

**Table 2:** Analysis of molecular variance AMOVA for the extended *12S* and concatenated *12S*+*16S*+*ND2* datasets of the mtDNA for *D*. *longispina* and two clades. The significances of Φ-statistics values were tested by a permutation test with 10 000 replicates.

Grouping criterion	Source of variation	df		% of variance		Φ-statistics	
		*12S*	con	*12S*	con	*12S*	con
Geographic areas	Among areas	7	‒	24.81	‒	Φ_SC_ = 0.282**	‒
	Among populations within area	18	‒	21.21	‒	Φ_ST_ = 0.460**	‒
	Within populations	187	‒	53.97	‒	Φ_CT_ = 0.248**	‒
Unstructured set	Among populations	25	‒	44.78	‒	Φ_ST_ = 0.448**	‒
	Within populations	187	‒	55.22	‒		
*D*. *longispina* clades	Among clades	1	1	73.39	71.77	Φ_SC_ = 0.213**	Φ_SC_ = 0.389**
	Among populations within clades	9	4	5.68	10.98	Φ_ST_ = 0.791**	Φ_ST_ = 0.827*
	Within populations	46	10	20.93	17.26	Φ_CT_ = 0.734**	Φ_CT_ = **0.718**

* *P* < 0.05

** *P* < 0.001

bold type—insignificant value, *P* > 0.05.

**Table 3:** Analysis of molecular variance AMOVA for the extended *12S* dataset of the mtDNA for *D*. *dentifera*. The significances of Φ-statistics values were tested by a permutation test with 10000 replicates.

Grouping criterion	Source of variation	df	% of variance	Φ-statistics
Geographic areas	Among areas	2	10.57	Φ_SC_ = 0.153*
	Among populations within areas	5	13.68	Φ_ST_ = 0.242*
	Within populations	79	75.75	Φ_CT_ = **0.106**
Unstructured set	Among populations	7	22.07	Φ_ST_ = 0.221*
	Within populations	79	77.93	

* *P* < 0.001

bold type—insignificant value, *P* > 0.05.

Evolutionary divergence (uncorrected *p*-distances) over the original *12S* sequences pair within and between studied species and clades of the *D*. *longispina* complex was high (7.3–12.5%, Table 4). Within certain species, the highest values for *p*-distances were found in *D*. *umbra*– 1.5%. As for genetic divergence between the geographical *D*. *longispina* populations, the uncorrected *p*-distances were found between populations belonging to divergent clades (S6 Table). The genetic distances obtained for *D*. *dentifera* haplogroups exhibited a significant divergence under pairwise comparison of the populations from the Baikal basin with the populations from Mongolia, China (Nepal) Canada and USA (S7 Table).

**Table 4:** Estimate of the evolutionary divergence (uncorrected *p*-distances, %) over the original *12S* sequences pair within and between studied species and clades of the *D*. *longispina* complex. Standard error estimates are shown above the diagonal.

Species	Within clade	*D*. *longispina* clade A	*D*. *longispina* clade B	*D*. *dentifera*	*D*. *turbinata*	*D*. *cucullata*	*D*. *galeata*	*D*. *umbra*	*D*. cf. *longispina*
*D*. *longispina* clade A	0.2±0.1	–	0.5	0.7	1.0	1.2	1.1	1.3	1.2
*D*. *longispina* clade B	0.9±0.2	2.2	–	0.7	1.0	1.2	1.1	1.2	1.3
*D*. *dentifera*	0.1±0.1	3.6	3.6	–	0.9	1.2	1.1	1.2	1.3
*D*. *turbinata*	0.1±0.1	7.0	7.2	6.8	–	1.3	1.2	1.2	1.3
*D*. *cucullata*	0.5±0.2	10.3	10.2	10.0	11.2	–	1.0	1.5	1.4
*D*. *galeata*	0.4±0.1	7.7	8.5	8.5	9.5	7.9	–	1.3	1.3
*D*. *umbra*	1.4±0.4	11.7	11.9	11.4	11.5	14.9	13.1	–	1.2
*D*. cf. *longispina*	0	9.4	10.3	10.7	10.9	12.6	11.3	11.1	–

**Fig 5:**
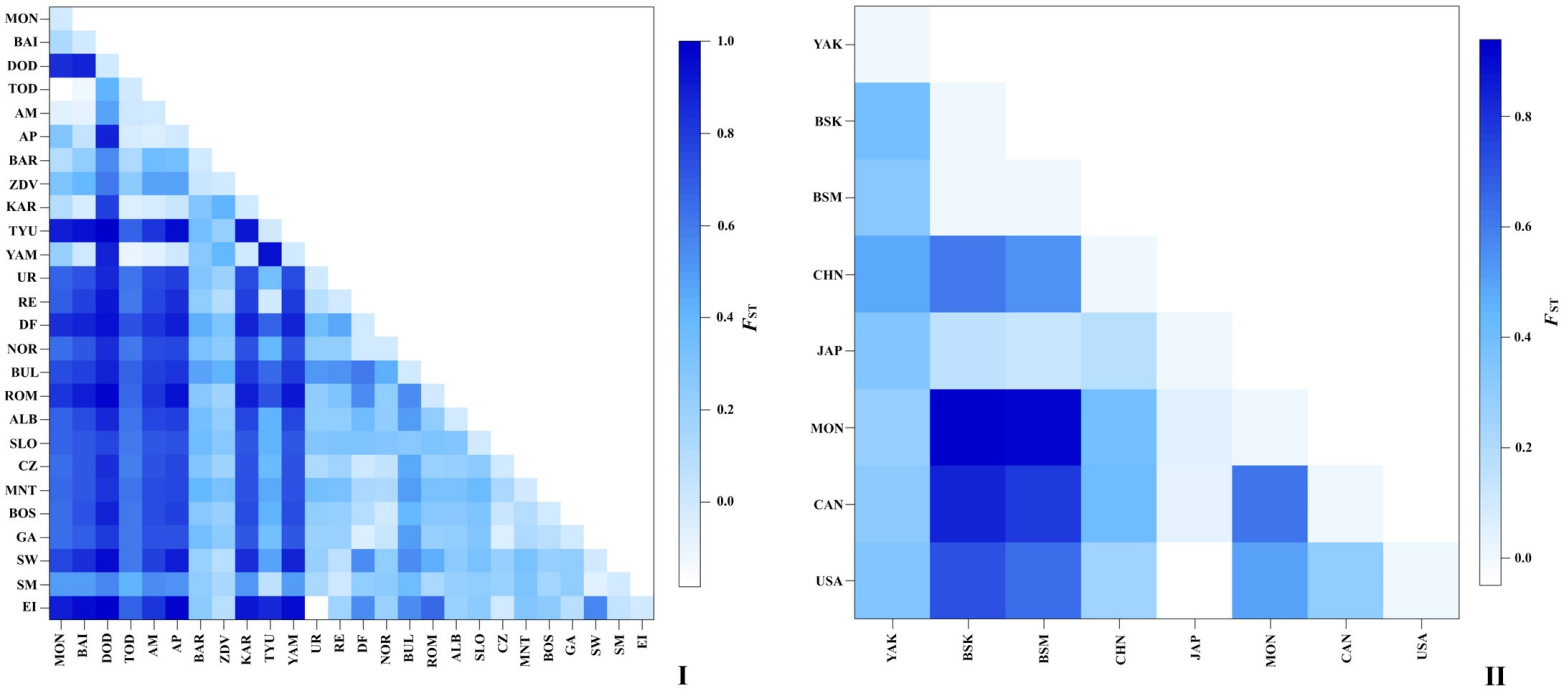
Graph of pairwise *F*_ST_ distance matrices between geographical populations of *D*. *longispina* (I) and *D*. *dentifera* (II). Codes are the same as in S3 and S4.

Pairwise *F*_ST_–values calculated between geographical populations of *D*. *longispina* indicated the occurrence of a high degree of genetic divergence between several of them. The highest value is detected, as expected, between population from Lake Dodot and all others, up to 1.0 (Fig 5I, S6 Table). For the *D*. *dentifera* populations, the highest *F*_ST_–values were found under pairwise comparison of the populations from the Baikal basin with the population from Mongolia, USA and Canada (Fig 5II, S6 Table).

### Neutrality tests

Most of the studied *Daphnia* species are characterized by negative values of Fu’s *F*_S_ and Tajima’s *D* tests (Table 5). Positive (but insignificant) value of Fu’s *F*_S_ for *D*. *umbra* was registered in the original *12S* dataset analysis, and for *D*. cf. *longispina* in the concatenated analysis. The positive (but insignificant) Tajima’s *D* values are found for *D*. *umbra* in the *12S* original dataset and for *D*. cf. *longispina* in the concatenated dataset analysis (Table 5). These neutrality tests estimated for the extended dataset are characterized by negative values for all *Daphnia* species and are significant for two *D*. *longispina* clades, *D*. *dentifera*, *D*. *galeata* and *D*. *cristata* species.

**Table 5:** The neutrality tests based on extended and original *12S* datasets and the concatenated *12S* + *16S* + *ND2* dataset of the mitochondrial DNA for species of the *D*. *longispina* complex.

Species/Clades	Fu’s *F*_S_			Tajima’s *D*		
	extended *12S*	original *12S*	concatenated	extended *12S*	original *12S*	concatenated
*D*. *longispina* clade A	-9.982**	-14.983**	-1.492	-2.309**	-2.398**	-1.322
*D*. *longispina* clade B	-257.959**	-11.596**	-0.537	-2.199**	-1.321	-0.608
*D*. *dentifera*	-71.153*	-1.539	-1.741	-2.225**	-1.580	-0.065
*D*. *turbinata*	-0.438	-0.438	‒	-0.275	-0.275	‒
*D*. *cucullata*	-8.457	-0.167	‒	-1.748	0.254	‒
*D*. *galeata*	-50.778*	-5.473**	-2.024*	-1.988*	-1.973*	-1.771
*D*. *umbra*	-1.579	2.815	‒	-0.271	0.775	‒
*D*. *lacustris*	-1.256	‒	‒	-0.829	‒	‒
*D*. *cristata*	-0.027**	‒	‒	-2.276**	‒	‒
*D*. cf. *longispina*	0	0	0.540	0	0	1.633

*P < 0.05;

**P < 0.01.

## Discussion

### Phylogeny of the *D*. *longispina* complex and originality of South Siberian taxa and clades

Some previous studies found that Southern Siberia, including Altai-Sayan region, was an important refugium for the terrestrial fauna [80, 81]. Moreover, it was critical for the human population survival in Asia duing the late Pleistocene cold phases, for example, there is evidence that this area was the sole refugium of humans in the region during Marine Isotopic Stage 4 [82, 83]. We found that it was also an important refugium for the Cladocera (and all freshwater fauna?) during the Pleistocene.

Our study of the genetic variability of the Asian *D*. *longispina* complex revealed unexpected diversity in the region. Specifically, we established the genetic uniqueness of *D*. *turbinata*, *D*. cf. *longispina* from Western Siberia and a Siberian clade A of *D*. *longispina* s. str. Southern Siberia was already found to be a source of locally distributed ancient phylogroups in *D*. *magna* [27]. *D*. *turbinata* is presently found only in the water bodies of Altai-Sayan Mountain System (including Mongolian Altai) (Fig 1, S1 Table). However, as with many montane species, the current range may represent but a small portion of a previously larger range.

*D*. *turbinata* is a sister group to the *D*. *longispina* ‒ *D*. *dentifera* clade. The genetic uniqueness of *D*. *turbinata* indicates that this species may be the oldest known “round helmeted” species of the *longispina* complex. Some discordances among topologies are related to the unresolved positions of Siberian *D*. *turbinata* and *D*. cf. *longispina* (as well as to the European *Daphnia* sp. from Berse). The phylogeny based on the larger concatenated mitochondrial dataset should be more reliable than the smaller datasets (provided that systematic biases are weak). The concatenated data tree has higher support values for its main branches, separate clades and internal divergent lineages. BI and ML analyses based on the concatenated mitochondrial dataset suggest the monophyly of a *D*. *longispina*–*D*. *dentifera*–*D*. *turbinata*–*D*. *galeata* clade.

*D*. cf. *longispina* from Western Siberia is not closely related to any other taxon of the *D*. *longispina* complex—no analysis supported its grouping with derived species in the clade. Moreover, a basal position of *D*. cf. *longispina* in the general phylogeny of the *D*. *longispina* complex is corroborated by the nuclear *ITS2* phylogenetic analysis [49]. To date, no morphological differences have been found between *D*. cf. *longispina* from Western Siberia and *D*. *longispina* s.str. This is unsurprising as parthenogenetic female cladocerans are often proposed to be subject to morphological stasis [84, 85, 86]. But, morphological differences may be found by comparing the adult males (presently unknown in *D*. cf. *longispina*), which are usually a more valuable source of diagnostic characters for cladocerans [12, 18], including the *D*. *longispina* complex [47]. *D*. *turbinata* and *D*. cf. *longispina*, basal taxa, may be regarded as phylogenetic relicts [87]. At the same time, populations of *D*. *umbra* and *D*. *turbinata* in the Siberian mountains and *D*. cf. *longispina* in Western Siberian lowlands are, most probably, the remains of pre-Pleistocene fauna that survived in Pleistocene refugia. In a sense these are "biogeographic relicts".

### Uniqueness of the *D*. *longispina* s. str. populations in Southern Siberia

A high level of the intra-population genetic variability has been detected for the *D*. *longispina* complex in different geographic regions [6, 8, 20, 37, 39, 88]. We detected many mitochondrial lineages within *D*. *longispina* s. str., and these lineages form two major geographic clades, presumably having different evolutionary history. A similar geographic clade association was detected for European and Siberian *ND2* haplotypes of "*D*. *rosea* s. lat." [30], which was the term that Ishida & Taylor [30] used for the *D*. *longispina* s. str.*/D*. *dentifera* clade of the present study. In present study, the divergences among lineages of *longispina* are reduced compared to [30] because we included mitochondrial genes that are relatively slowly evolving (i.e., 12S rRNA). Haplotypes of clade B have a wide geographic distribution—from Western Europe to the Yenisei River basin. This clade is strongly genetically structured, i.e. includes several subclades. On the one hand, a high degree of genetic polymorphism could be a consequence of secondary contact of heterogeneous populations and genetically divergent lineages (i.e. contact from separate glacial refugia). Such an explanation of the high genetic heterogeneity was previously accepted for several cladoceran taxa including *D*. *longispina* [13, 20, 30, 33, 36]. On the other hand, such a high genetic divergence could be the result of ongoing cryptic speciation [26]. It was demonstrated recently that divergent mitochondrial lineages of *D*. *longispina* may sometimes have a hybrid origin [16]. New detailed studies are necessary for a final understanding of the cases of such high genetic polymorphism in the clade B which is associated with weak morphological differentiation. But note that the only a single haplotype from the clade B found in mountains of Southern Siberia.

In contrast to the pattern found in clade B, haplotypes from the South Siberian mountains form the bulk of clade A. Divergent haplotypes of *D*. *longispina* from Lake Dodot and the lakes of the Altai Mountains, which compose the majority of clade A, could be relicts of an ancient fauna. Long-term geographic isolation may have led to their strong genetic divergence—as a result a unique haplotypic complex is now present. According to the *F*_ST_ values, gene flow between populations forming different clades of *D*. *longispina* is very limited both on a regional (the original *12S* dataset), and a pan-Eurasian (the extended *12S* dataset) scale. Limited gene flow between populations makes their differentiation stronger because the frequency of unique haplotypes is increasing with time. In general, a high level of intra-population variability in combination with a strong inter-population genetic differentiation agrees well with the logic of the "monopolization hypothesis" [20, 89].

The geology and climate of Siberia during the Late Pleistocene could be one reason for small genetic distances between populations of *D*. *longispina* from water bodies of the Ural Mountains, the Lake Chany basin and Western Europe. In the Late Pleistocene, large North Siberian rivers were dammed by an ice sheet, huge periglacial lakes were formed in northern portion of Eurasia, merging with each other and uniting the whole Western and Eastern hydrological systems (including the Volga, Ob; and Yenisei basins) from the Alps to territory of recent Yakutia [90]. Mansiysoe Lake existed at that time in Western Siberia (i.e. covering the region of recent Lake Chany).

At the same time, glaciation was only partial and patchy in the Altai Mountains, Sayan Mountains and Eastern Siberian lowlands [90, 91]. Mixing of the lineages within the clade B of *D*. *longispina* may have occurred during this time, while lineages of clade A were isolated in refugia of the Altai and Sayan mountains. During warmer phases of the Pleistocene, the northern drainage to the Polar Ocean was restored [90, 92], and resting stages of the daphniids would have had the opportunity to disperse from the refugia in the Altai and Sayans towards the lower reaches of Siberian rivers. This geological scenario may explain the appearance of clade A in the Lake Chany basin and the Yamalo-Nenets Area (lower reaches of the Ob River). Alternatively, clade A may have come from another area where they subsequently disappeared.

Changes of climate and, as a result, hydrology of Siberian rivers took place many times during the Pleistocene. Recent populations of *D*. *longispina*, forming clade B, have appeared as a result of multiple secondary contacts between partially and temporarily isolated mitochondrial lineages on a large geographic scale. In contrast, populations of clade A were isolated for a long period adding to genetic differentiation.

Previously, we proposed that the sister taxa, *D*. *dentifera* and *D*. *longispina*, were vicariant species with a transition zone in the Yenisei River basin. At the time, *D*. *longispina* was undetected in Eastern Eurasia, while *D*. *dentifera* populations were dominant in Eastern Eurasia [48] and the western Nearctic [30]. The present study agrees with this hypothesis: but now *D*. *dentifera* is also found Yakutia (the Lena River basin). It is remarkable that in China, *D*. *dentifera* is a dominant species, while *D*. *longispina* is very rare [35, 37, 38, 93, 94]. Unfortunately, the existence of a transitional zone between the two taxa was not discussed based on the records from China. East-west longitudinal differentiation, with a transition zone between western and eastern taxa or phylogroups in the Yenisey River basin, has been demonstrated for other cladoceran genera [26, 43, 95, 96]. A role for interspecific interactions with *D*. *longispina* limiting the western expansion of *D*. *dentifera* is supported by the same postglacial expansion of *D*. *dentifera* eastward (in the absence of *D*. *longispina)* across Beringia to much of the Nearctic [30].

Previous authors pointed out several times that *D*. *longispina*/*D*. *dentifera* and *D*. *galeata* have differing preferences for trophic status and hydrological traits with *D*. *galeata* being associated with more nutrient rich waters [36, 39, 97, 98]. Our observations partially corroborate this view. Indeed, in the pelagic zone of the Todzha Depression *D*. *galeata* dominated, while *D*. *longispina* occurred only in shallow bays or small ponds [99]. But, at the same time, *D*. *longispina* and *D*. *galeata* appear to co-occur in small temporary lakes in the Lake Chany basin (keeping in mind that our methods may fail to detect hybrid products). Differing ecological conditions may lead to adaptations that increase divergences between geographically distant populations [100, 101, 102].

### Demographic history

Most mitochondrial clades of *D*. *longispina* and other species demonstrate statistically significant negative values of neutrality tests (Fu’s *F*_S_ and Tajima’s *D*). Such values are usually interpreted as consequences of three processes: (1) recent (probably, post-glacial) spatial expansion, (2) negative selection and/or (3) genetic hitchhiking (when an allele changes frequency not because it itself is under natural selection, but because it is near another gene that is undergoing a selective sweep and that is on the same DNA chain) [78, 79]. At the same time, some neutrality tests for *D*. *umbra* and *D*. cf. *longispina* gave positive (although non-significant) values. Such differences are likely consequences of different sampling efforts.

Keeping in mind the aforementioned wide geographic distribution of the clade B haplotypes, high haplotype number, high vales of haplotypic (*H*_d_) and nucleotide (π) diversity and high genetic divergence (*p*-distances) between individuals, we can propose that the results of neutrality tests (i.e. an unusually high Fu’s *F*_S_ value) confirm a recent spatial expansion of these haplotypes. High values of *H*_d_ and π, most probably, are consequences of a mixing of historically heterogenous and geographically differentiated populations of the clade B, instead of existence of a stable population with large effective size [103]. The bimodal structure of the mismatch distribution diagram for the clade B [48] could also be explained by an additional internal structure instead of an equilibrium state.

Negative and significant values of Fu’s *F*_S_ and Tajima’s *D*, as well as a star-like shape of the network, are consistent with a recent expansion of the clade A. But a low number of haplotypes, high values of *H*_d_ and lower vales of π and *p*-distances for the clade A (as compared with clade B) most probably reflect a colonization of this region by one or few genetically depauperate populations of *D*. *longispina*. The studied populations may have originated recently from an ancestral population with lower effective population size. This time was sufficient for the population to restore a haplotypic diversity, but not nucleotide diversity [103]. As haplotypes of clade A dominate in the Altai and Sayan water bodies, we can assume that the ancestral population survived during Pleistocene in a mountain refugium. Its effective size was relatively large, as the unique haplotypic structure of *D*. *longispina* was retained. Founder effect in the mountain water bodies of Altai and Sayan appeared strong, as a result a rate of the clade B haplotypes in this region is minute.

The time of expansion was probably different for clades A and B [48], even keeping errors in divergence estimations in mind [104]. An earlier differentiation of clade B is supported by the extremely high negative values of Fu’s *F*_S_ test and a wider geographic distribution of haplotypes, although recent expansion of this clade has waned or stopped. Strong genetic differentiation and structure of clade B support recent or ongoing divergence of its internal groups due to their local adaptations after reaching an equilibrium state (see the multimodal mismatch distribution [48]). The subsequent divergence of clade B has been discussed concerning European populations of *D*. *longispina* [39].

There are several factors preventing a further geographic expansion of clade B. Its distribution towards the east may be difficult due to occupation of the available water bodies by a potentially competing taxon, *D*. *dentifera*. The latter is characteristic of a high polymorphism level (high haplotype number, high values of *H*_d_ and π) and negative values of Fu’s *F*_S_ and Tajima’s *D* tests. Penetration of clade B to the water bodies of the Altai and Sayan mountains is difficult as they are occupied by the clade A populations. Most probably, some difficulties to occupy new water bodies may involve *D*. *galeata*, a widely distributed taxon in lacustrine systems [35, 38, 105]. Interestingly some the haplotypes of *D*. *galeata* also form divergent regional clades in Siberia.

There is little evidence for spatial expansion in *D*. *turbinata*. Fu’s *F*_S_ and Tajima’s *D* tests, had a negative sign that lacked statistical significance. These values together with low intra-species genetic divergence and a relatively low level of genetic polymorphism may be due to recovery from a bottleneck event (possibly the last Pleistocene glaciation) [103]. The low values of *H*_d_ and π in the Asian mountain endemic (*D*. *turbinata*) and Western Siberian endemic (*D*. cf. *longispina*) are similar to those of the European relict, *D*. *lacustris*. Most probably, these taxa are relicts of a pre-glacial fauna. Cladocerans are an ancient group [106, 107]. However, some lineages differentiated before the Pleistocene [28, 95], while others differentiated in a rapid post-glacial manner [19].

## Conclusions

Our phylogeny of the *D*. *longispina* complex supports the monophyly of *D*. *longispina*–*D*. *dentifera*–*D*. *turbinata*–*D*. *cucullata*–*D*. *galeata* clade, while *D*. cf. *longispina* represents an earlier derived taxon of the D. *longispina* complex. Our analysis of the genetic polymorphism of the mitochondrial DNA revealed a high level of population genetic structure within each taxon. The highest divergence is characteristic of *D*. *longispina* having two major geographic clades. The geographic range of several haplotypes is limited to the Altai-Sayan region. Clade B did not penetrate mountain water bodies that clade A colonized. Further expansion of the clade B east appears limited by the counter expansion of *D*. *dentifera*. The zone of their secondary contact is located in the Yenisei-Baikal region. Southern Siberian endemics, mountain *D*. *turbinata*, *D*. cf. *longispina* (which is present now only in the Lake Chany basin), and *D*. *umbra* (for which populations in Southern Siberia are disjunct from Arctic populations) appear to have passed through a prolonged "bottleneck". These populations are relicts of pre-glacial times. A complicated geological and climatic history of the Altai-Sayan mountain system promotes forming an original species and haplotypic composition of the D. *longispina* complex in this region.

## Supporting information

S1 Table List of the original *Daphnia* sequences (*12S*, *16S* and *ND2*) of mitochondrial DNA used for the genetic analyses and phylogeny reconstruction (abbreviation, region, locality, geographical position). * newly obtained sequences (73); specimens sequenced on three mitochondrial genes are marked by green color.
(XLS)

S2 Table List of the *Daphnia 12S* sequences of mitochondrial DNA obtained from the GenBank database and used in the extended dataset for the genetic analyses and phylogeny reconstruction. (XLS)

S3 Table List of the geographical areas used for the AMOVA and *F*_ST_ analyses grouped on the extended *12S* dataset for *D*. *longispina*. (DOCX)

S4 Table List of the geographical areas used for the AMOVA and *F*_ST_ analyses grouped on the extended *12S* dataset for *D*. *dentifera*. (DOCX)

S5 Table Polymorphism of the mtDNA based on the extended *12S* dataset for species of the *D*. *longispina* complex. Abbreviations: *n*, number of sequenced *Daphnia* individuals; *S*, number of polymorphic sites; *h*, number of haplotypes; *H*_d_, haplotype diversity; **π,** nucleotide diversity; st.d., standard deviation.
(DOC)

S6 Table Uncorrected *p*-distances (%, below the diagonal) and pairwise *F*_ST_ (above the diagonal) based on the extended *12S* dataset between *D*. *longispina* geographical populations. The analysis involved 213 nucleotide sequences. The abbreviations (##) correspond to same in Table S4.
(DOC)

S7 Table Uncorrected *p*-distances (%, below the diagonal) and pairwise *F*_ST_ (above the diagonal) based on the extended *12S* dataset between *D*. *dentifera* geographical populations. The analysis involved 87 nucleotide sequences. The abbreviations (##) correspond to same in S1 Fig.
(DOC)

S1 Fig Bayesian phylogenetic consensus tree for *D*. *longispina* based on the extended *12S* dataset. Bayesian posterior probabilities BI and bootstrap values from ML analysis above 75% expressed as a percentage are indicated for each significant node. Scale is given in expected substitutions per site. Color of geometric symbol for original sequences corresponds to geographical sampling areas: dark blue, Kamchatka Peninsula; green, Yakutia; brown, Mongolia; bright-green, Baikal basin and Transbaikalia; red, the Yenisei basin; turquoise, Altai Mountains; yellow, Altai plain areas; pink, middle and lower reach of the Ob-Irtysh basin; grey, Ural; white, Eastern and Central Europe. Figure of geometric symbol identify species: solid circle, *D*. *longispina*; open circle, *D*. *dentifera*; triangle, *D*. *cucullata*; reverse triangle, *D*. *turbinata*; solid square, *D*. *galeata*; open square, *D*. cf. *longispina*; diamond, *D*. *umbra*; star, *D*. *cristata*. *D*. *longispina* clade A and *D*. *turbinata* are highlighted in red and brown, respectively. Arrow indicates sequence from type locality of *D*. *longispina*, Denmark.
(TIF)

S2 Fig Bayesian phylogenetic tree for species of the *D*. *longispina* complex based on the original *12S* dataset. Bayesian posterior probabilities BI above 72% expressed as a percentage is indicated for each significant node. Scale is given in expected substitutions per site. Colored geometric symbols are the same as in S1 Fig.
(TIF)
