# Supplementary material for: Unexpected endemism in the Daphnia longispina complex (Crustacea: Cladocera) in Southern Siberia
Source: PLoS One. 2019 Sep 3;14(9):e0221527. doi: 10.1371/journal.pone.0221527 (PMC6719860; doi:10.1371/journal.pone.0221527)
Supplement: S3 Table — (DOCX) [file pone.0221527.s003.docx]

**S3 Table. List of the geographical areas used for the AMOVA and *F*_ST_ analyses grouped on the extended *12S* dataset for *D. longispina*.**

| **##** | **Geographical area** | [**Abbreviation**](https://www.multitran.ru/c/m.exe?t=428_1_2&s1=%E0%E1%E1%F0%E5%E2%E8%E0%F2%F3%F0%E0) | **Groups** |
| --- | --- | --- | --- |
| 1. | Mongolia | MON | 1 |
| 2. | Baikal | BAI | 1 |
| 3. | Dodot Lake | DOD | 2 |
| 4. | Todzha Depression | TOD | 2 |
| 5. | Altai Mountains | AM | 2 |
| 6. | Altai (plain) | AP | 3 |
| 7. | Barabinsk | BAR | 3 |
| 8. | Zdvinsk | ZDV | 3 |
| 9. | Kargat | KAR | 3 |
| 10. | Tyumen | TYU | 4 |
| 11. | Yamal | YAM | 4 |
| 12. | Ural | UR | 4 |
| 13. | Russian Europe | RE | 5 |
| 14. | Denmark + Finland | DF | 5 |
| 15. | Norway | NOR | 5 |
| 16. | Bulgaria | BUL | 6 |
| 17. | Romania | ROM | 6 |
| 18. | Albania | ALB | 6 |
| 19. | Slovenia | SLO | 6 |
| 20. | Czech | CZ | 5 |
| 21. | Montenegro | MNT | 6 |
| 22. | Bosnia | BOS | 6 |
| 23. | Germany + Austria | GA | 7 |
| 24. | Switzerland | SW | 7 |
| 25. | Spain + Morocco | SM | 8 |
| 26. | Ethiopia + Israel | EI | 8 |
