# Supplementary material for: Unexpected endemism in the Daphnia longispina complex (Crustacea: Cladocera) in Southern Siberia
Source: PLoS One. 2019 Sep 3;14(9):e0221527. doi: 10.1371/journal.pone.0221527 (PMC6719860; doi:10.1371/journal.pone.0221527)
Supplement: S4 Table — (DOCX) [file pone.0221527.s004.docx]

**S4 Table. List of the geographical areas used for the AMOVA and *F*_ST_ analyses grouped on the extended *12S* dataset for *D. dentifera*.**

| **##** | **Geographical area** | **Abbreviation** | **Groups** |
| --- | --- | --- | --- |
| 1. | Yakutia | YAK | 1 |
| 2. | Baikal: Srednee Kedrovoye | BSK | 1 |
| 3. | Baikal: Sagan-Moryan | BSM | 1 |
| 4. | Mongolia | MON | 2 |
| 5. | China + Nepal | CHN | 2 |
| 6. | Japan | JAP | 2 |
| 7. | Canada | CAN | 3 |
| 8. | USA | USA | 3 |
