# Supplementary material for: Unexpected endemism in the Daphnia longispina complex (Crustacea: Cladocera) in Southern Siberia
Source: PLoS One. 2019 Sep 3;14(9):e0221527. doi: 10.1371/journal.pone.0221527 (PMC6719860; doi:10.1371/journal.pone.0221527)
Supplement: S5 Table — Abbreviations: n, number of sequenced Daphnia individuals; S, number of polymorphic sites; h, number of haplotypes; Hd, haplotype diversity; π, nucleotide diversity; st.d., standard deviation. (DOC) [file pone.0221527.s005.doc]

***S5 Table. Polymorphism of the mtDNA based on the extended 12S dataset for species of the D. longispina complex****. Abbreviations: n, number of sequenced Daphnia individuals; S, number of polymorphic sites; h, number of haplotypes; H_d_, haplotype diversity;* ***π,*** *nucleotide diversity;**st.d., standard deviation.*

| **Species** | ***n*** | ***h*** | ***S*** | ***H*_d_ ± st.d.** | **π ± st.d.** |
| --- | --- | --- | --- | --- | --- |
| *D. longispina* clade A  *D. longispina* clade B  *D. dentifera*  *D. turbinata*  *D. cucullata*  *D. galeata*  *D. umbra*  *D. lacustris*  *D. cristata* | 44  166  87  7  18  93  18  12  16 | 13  138  68  3  13  47  11  4  5 | 18  114  116  3  17  44  22  3  14 | 0.701±0.068  0.997±0.001  0.978±0.010  0.667±0.0255  0.948±0.039  0.920±0.023  0.935±0.035  0.561±0.154  0.450±0.151 | 0.0023±0.0005  0.0125±0.0005  0.0164±0.0017  0.0015±0.0005  0.0053±0.000  0.0068±0.0006  0.0118±0.0009  0.0015±0.0005  0.0034±0.0021 |
