# Supplementary material for: Unexpected endemism in the Daphnia longispina complex (Crustacea: Cladocera) in Southern Siberia
Source: PLoS One. 2019 Sep 3;14(9):e0221527. doi: 10.1371/journal.pone.0221527 (PMC6719860; doi:10.1371/journal.pone.0221527)
Supplement: S6 Table — The analysis involved 213 nucleotide sequences. The abbreviations (##) correspond to same in Table S4. (DOC) [file pone.0221527.s006.doc]

**S6 Table. Uncorrected *p*-distances (%, below the diagonal) and pairwise *F*_ST_ (above the diagonal) based on the extended *12S* dataset between *D. longispina* geographical populations**. The analysis involved 213 nucleotide sequences. The abbreviations (##) correspond to same in Table S3.

| **##** | **UR** | **RE** | **YAM** | **SM** | **CZ** | **DF** | **GA** | **NOR** | **BUL** | **BOS** | **MNT** | **ROM** | **ALB** | **SLO** | **EI** | **SW** | **MON** | **TOD** | **BAI** | **TYU** | **AP** | **AM** | **KAR** | **ZDV** | **BAR** | **DOD** |
| --- | --- | --- | --- | --- | --- | --- | --- | --- | --- | --- | --- | --- | --- | --- | --- | --- | --- | --- | --- | --- | --- | --- | --- | --- | --- | --- |
| **UR** | ‒ | 0.8 | 0.7* | 0.1* | 0.1* | 0.3* | 0.2* | 0.2* | 0.5* | 0.2* | 0.3* | 0.2 | 0.2* | 0.3* | -0.2 | 0.2 | 0.7* | 0.6* | 0.7* | 0.3 | 0.8* | 0.7* | 0.7* | 0.2 | 0.3* | 0.8* |
| **RE** | 0.8 | ‒ | 0.8* | 0 | 0.2* | 0.4* | 0.2* | 0.2* | 0.5* | 0.2* | 0.3* | 0.3 | 0.2* | 0.3* | 0.2 | 0.1 | 0.7 | 0.6* | 0.8 | 0 | 0.8* | 0.8* | 0.8* | 0.1 | 0.2 | 0.9* |
| **YAM** | 2.1 | 2.0 | ‒ | 0.5* | 0.7* | 0.9* | 0.7* | 0.7* | 0.8* | 0.7* | 0.7* | 0.9 | 0.8* | 0.7* | 1.0 | 0.9 | 0.2 | -0.1 | 0 | 0.9* | 0 | 0 | 0 | 0.4 | 0.3 | 0.9* |
| **SM** | 1.2 | 1.1 | 2.0 | ‒ | 0.2* | 0.2* | 0.2 | 0.2* | 0.4* | 0.2* | 0.3* | 0.1* | 0.2* | 0.2* | 0 | -0.1 | 0.5* | 0.4* | 0.5* | 0.1 | 0.5* | 0.5* | 0.5* | 0.1* | 0.2* | 0.6* |
| **CZ** | 0.9 | 1.1 | 2.2 | 1.4 | ‒ | 0 | 0 | 0 | 0.4* | 0 | 0.1* | 0.2 | 0.2* | 0.2* | 0 | 0.2 | 0.6* | 0.6* | 0.7* | 0.4 | 0.8* | 0.7* | 0.7* | 0.2* | 0.3* | 0.9* |
| **DF** | 0.7 | 0.9 | 2.1 | 1.2 | 0.6 | ‒ | 0 | 0 | 0.6* | 0.1 | 0.1 | 0.5* | 0.3* | 0.3* | 0.5* | 0.5* | 0.8* | 0.7* | 0.9* | 0.7* | 0.9* | 0.8* | 0.9* | 0.3* | 0.4* | 0.9* |
| **GA** | 0.1 | 1.1 | 2.4 | 1.5 | 0.9 | 0.6 | ‒ | 0 | 0.5* | 0.1 | 0.1* | 0.2* | 0.2* | 0.3* | 0.1 | 0.2* | 0.6* | 0.6* | 0.7* | 0.3* | 0.7* | 0.7* | 0.7* | 0.2* | 0.3* | 0.8* |
| **NOR** | 0.1 | 1.1 | 2.3 | 1.5 | 0.9 | 0.6 | 1.0 | ‒ | 0.4* | 0 | 0.1* | 0.2 | 0.2* | 0.3* | 0.2 | 0.2 | 0.6* | 0.6* | 0.7* | 0.4* | 0.8* | 0.7* | 0.7* | 0.2* | 0.3* | 0.8* |
| **BUL** | 1.3 | 1.4 | 2.3 | 1.7 | 1.4 | 1.2 | 1.6 | 1.3 | ‒ | 0.4* | 0.5* | 0.5* | 0.5* | 0.3* | 0.5* | 0.5* | 0.7* | 0.7* | 0.8* | 0.6* | 0.8* | 0.8* | 0.8* | 0.4* | 0.5* | 0.9* |
| **BOS** | 1.1 | 1.2 | 2.3 | 1.4 | 1.0 | 0.7 | 1.1 | 0.9 | 1.3 | ‒ | 0.1 | 0.3 | 0.3* | 0.3* | 0.2 | 0.2 | 0.6 | 0.6* | 0.7* | 0.4* | 0.8* | 0.7* | 0.7* | 0.2* | 0.3* | 0.9* |
| **MNT** | 1.3 | 1.4 | 2.6 | 1.7 | 1.1 | 0.8 | 1.1 | 1.1 | 1.6 | 1.1 | ‒ | 0.3 | 0.3* | 0.4* | 0.3 | 0.3 | 0.6* | 0.6* | 0.7* | 0.4* | 0.8* | 0.7* | 0.7* | 0.3* | 0.4* | 0.8* |
| **ROM** | 0.8 | 0.9 | 2.1 | 1.3 | 1.0 | 0.7 | 1.1 | 1.0 | 1.3 | 1.1 | 1.3 | ‒ | 0.2 | 0.3* | 0.7 | 0.4 | 0.8 | 0.7* | 0.9 | 0.7 | 0.9* | 0.8* | 0.9 | 0.2 | 0.3 | 1.0* |
| **ALB** | 0.9 | 1.1 | 2.3 | 1.4 | 1.1 | 0.9 | 1.2 | 1.1 | 1.4 | 1.2 | 1.4 | 0.9 | ‒ | 0.3* | 0.2 | 0.2 | 0.7* | 0.6* | 0.7* | 0.4* | 0.8* | 0.8* | 0.8* | 0.2* | 0.3* | 0.9* |
| **SLO** | 0.9 | 1.1 | 2.0 | 1.3 | 1.1 | 0.8 | 1.1 | 1.1 | 0.9 | 1.1 | 1.3 | 0.9 | 1.1 | ‒ | 0.2 | 0.3* | 0.7* | 0.6* | 0.7* | 0.4* | 0.7* | 0.7* | 0.7* | 0.3* | 0.4* | 0.8* |
| **EI** | 0.3 | 0.6 | 2.0 | 1.0 | 0.6 | 0.5 | 0.8 | 0.8 | 1.1 | 0.9 | 1.1 | 0.6 | 0.7 | 0.8 | ‒ | 0.5 | 0.9 | 0.7* | 0.9 | 0.9 | 1.0* | 0.8* | 0.9 | 0.1 | 0.2 | 1.0* |
| **SW** | 0.8 | 0.8 | 1.9 | 1.0 | 1.1 | 0.8 | 1.2 | 1.1 | 1.4 | 1.1 | 1.4 | 0.9 | 1.0 | 1.0 | 0.7 | ‒ | 0.8 | 0.6 | 0.8 | 0.5 | 0.9* | 0.8* | 0.8 | 0.1 | 0.2 | 0.9* |
| **MON** | 2.2 | 2.2 | 0.3 | 2.2 | 2.2 | 2.1 | 2.4 | 2.4 | 2.3 | 2.3 | 2.6 | 2.2 | 2.3 | 2.0 | 2.0 | 2.1 | ‒ | -0.2 | 0.1 | 0.9 | 0.3 | 0 | 0.1 | 0.3 | 0.1 | 0.8* |
| **TOD** | 1.9 | 1.9 | 0.4 | 1.9 | 2.0 | 1.9 | 2.2 | 2.1 | 2.1 | 2.1 | 2.4 | 2.0 | 2.1 | 1.8 | 1.8 | 1.7 | 0.5 | ‒ | -0.1 | 0.7* | 0 | 0 | 0 | 0.2 | 0.1 | 0.4* |
| **BAI** | 2.2 | 2.1 | 0.1 | 2.1 | 2.3 | 2.2 | 2.5 | 2.4 | 2.4 | 2.4 | 2.6 | 2.3 | 2.4 | 2.1 | 2.1 | 2.0 | 0.3 | 0.4 | ‒ | 0.9 | 0 | 0 | 0 | 0.4 | 0.2 | 0.9* |
| **TYU** | 0.7 | 0.5 | 1.8 | 1.0 | 1.0 | 0.8 | 1.1 | 1.1 | 1.4 | 1.1 | 1.3 | 0.9 | 1.0 | 1.0 | 0.7 | 0.6 | 2.1 | 1.7 | 1.9 | ‒ | 0.9* | 0.8* | 0.9* | 0.2* | 0.3* | 1.0* |
| **AP** | 2.2 | 2.1 | 0.1 | 2.1 | 2.3 | 2.2 | 2.4 | 2.4 | 2.4 | 2.3 | 2.6 | 2.2 | 2.4 | 2.1 | 2.0 | 1.9 | 0.2 | 0.4 | 0.1 | 1.9 | ‒ | 0 | 0 | 0.5 | 0.3* | 0.9* |
| **AM** | 2.3 | 2.3 | 0.3 | 2.3 | 2.4 | 2.3 | 2.6 | 2.5 | 2.5 | 2.5 | 2.8 | 2.3 | 2.5 | 2.2 | 2.2 | 2.1 | 0.4 | 0.6 | 0.3 | 2.1 | 0.3 | ‒ | 0 | 0.5* | 0.4* | 0.5* |
| **KAR** | 2.2 | 2.1 | 0.2 | 2.2 | 2.3 | 2.2 | 2.5 | 2.5 | 2.4 | 2.4 | 2.7 | 2.3 | 2.4 | 2.1 | 2.0 | 2.0 | 0.3 | 0.5 | 0.2 | 2.0 | 0.1 | 0.3 | ‒ | 0.5 | 0.3* | 0.8* |
| **ZDV** | 1.1 | 1.2 | 1.3 | 1.4 | 1.3 | 1.1 | 1.4 | 1.4 | 1.6 | 1.4 | 1.6 | 1.2 | 1.3 | 1.2 | 1.0 | 1.1 | 1.4 | 1.3 | 1.4 | 1.0 | 1.4 | 1.6 | 1.4 | ‒ | 0 | 0.6* |
| **BAR** | 1.4 | 1.5 | 1.1 | 1.7 | 1.6 | 1.5 | 1.7 | 1.7 | 1.8 | 1.7 | 2.0 | 1.5 | 1.7 | 1.5 | 1.3 | 1.4 | 1.2 | 1.2 | 1.2 | 1.4 | 1.2 | 1.3 | 1.2 | 1.3 | ‒ | 0.5* |
| **DOD** | 2.3 | 2.3 | 0.3 | 2.3 | 2.4 | 2.3 | 2.6 | 2.6 | 2.5 | 2.5 | 2.8 | 2.4 | 2.5 | 2.2 | 2.2 | 2.1 | 0.4 | 0.6 | 0.3 | 2.1 | 0.2 | 0.4 | 0.3 | 1.6 | 1.3 | ‒ |

**P* < 0.05
