# Supplementary material for: Unexpected endemism in the Daphnia longispina complex (Crustacea: Cladocera) in Southern Siberia
Source: PLoS One. 2019 Sep 3;14(9):e0221527. doi: 10.1371/journal.pone.0221527 (PMC6719860; doi:10.1371/journal.pone.0221527)
Supplement: S7 Table — The analysis involved 87 nucleotide sequences. The abbreviations (##) correspond to same in S1 Fig. (DOC) [file pone.0221527.s007.doc]

**S7 Table. Uncorrected *p*-distances (%, below the diagonal) and pairwise *F*_ST_ (above the diagonal) based on the extended *12S* dataset between *D. dentifera* geographical populations. The analysis involved 87 nucleotide sequences.** The abbreviations (##) correspond to same in S4 Table. **P* < 0.05.

| **##** | **YAK** | **BSK** | **BSM** | **MON** | **CHN** | **JAP** | **CAN** | **USA** |
| --- | --- | --- | --- | --- | --- | --- | --- | --- |
| **YAK** | ‒ | 0.4* | 0.3* | 0.3 | 0.5* | 0.3* | 0.3 | 0.4* |
| **BSK** | 1.4 | ‒ | 0.3 | 0.6* | 0.6* | 0.2* | 0.8* | 0.7* |
| **BSM** | 1.4 | 0.0 | ‒ | 0.9* | 0.6* | 0.1* | 0.8* | 0.6* |
| **MON** | 1.9 | 0.5 | 0.5 | ‒ | 0.4 | 0.1 | 0.6 | 0.5 |
| **CHN** | 2.9 | 1.5 | 1.5 | 1.6 | ‒ | 0.2* | 0.4* | 0.3* |
| **JAP** | 2.8 | 1.4 | 1.4 | 1.6 | 2.1 | ‒ | 0.0 | 0.0 |
| **CAN** | 2.0 | 0.6 | 0.6 | 1.1 | 1.7 | 1.5 | ‒ | 0.3 |
| **USA** | 1.9 | 0.5 | 0.5 | 0.9 | 1.4 | 1.4 | 0.7 | ‒ |
