# Supplementary figures and images for: Unexpected endemism in the Daphnia longispina complex (Crustacea: Cladocera) in Southern Siberia
Source: PLoS One. 2019 Sep 3;14(9):e0221527. doi: 10.1371/journal.pone.0221527 (PMC6719860; doi:10.1371/journal.pone.0221527)

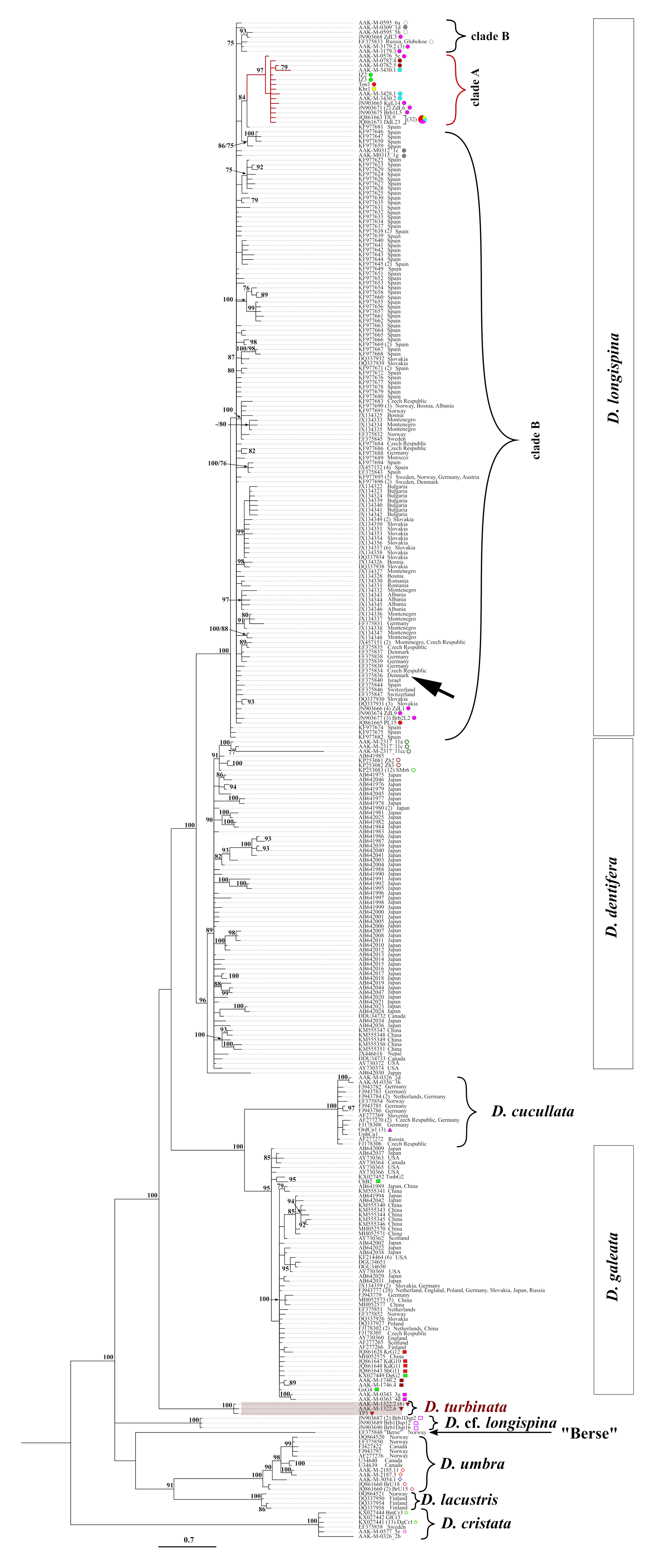

Supplement: S1 Fig — Bayesian posterior probabilities BI and bootstrap values from ML analysis above 75% expressed as a percentage are indicated for each significant node. Scale is given in expected substitutions per site. Color of geometric symbol for original sequences corresponds to geographical sampling areas: dark blue, Kamchatka Peninsula; green, Yakutia; brown, Mongolia; bright-green, Baikal basin and Transbaikalia; red, the Yenisei basin; turquoise, Altai Mountains; yellow, Altai plain areas; pink, middle and lower reach of the Ob-Irtysh basin; grey, Ural; white, Eastern and Central Europe. Figure of geometric symbol identify species: solid circle, D. longispina; open circle, D. dentifera; triangle, D. cucullata; reverse triangle, D. turbinata; solid square, D. galeata; open square, D. cf. longispina; diamond, D. umbra; star, D. cristata. D. longispina clade A and D. turbinata are highlighted in red and brown, respectively. Arrow indicates sequence from type locality of D. longispina, Denmark. (TIF) [file pone.0221527.s008.tif]

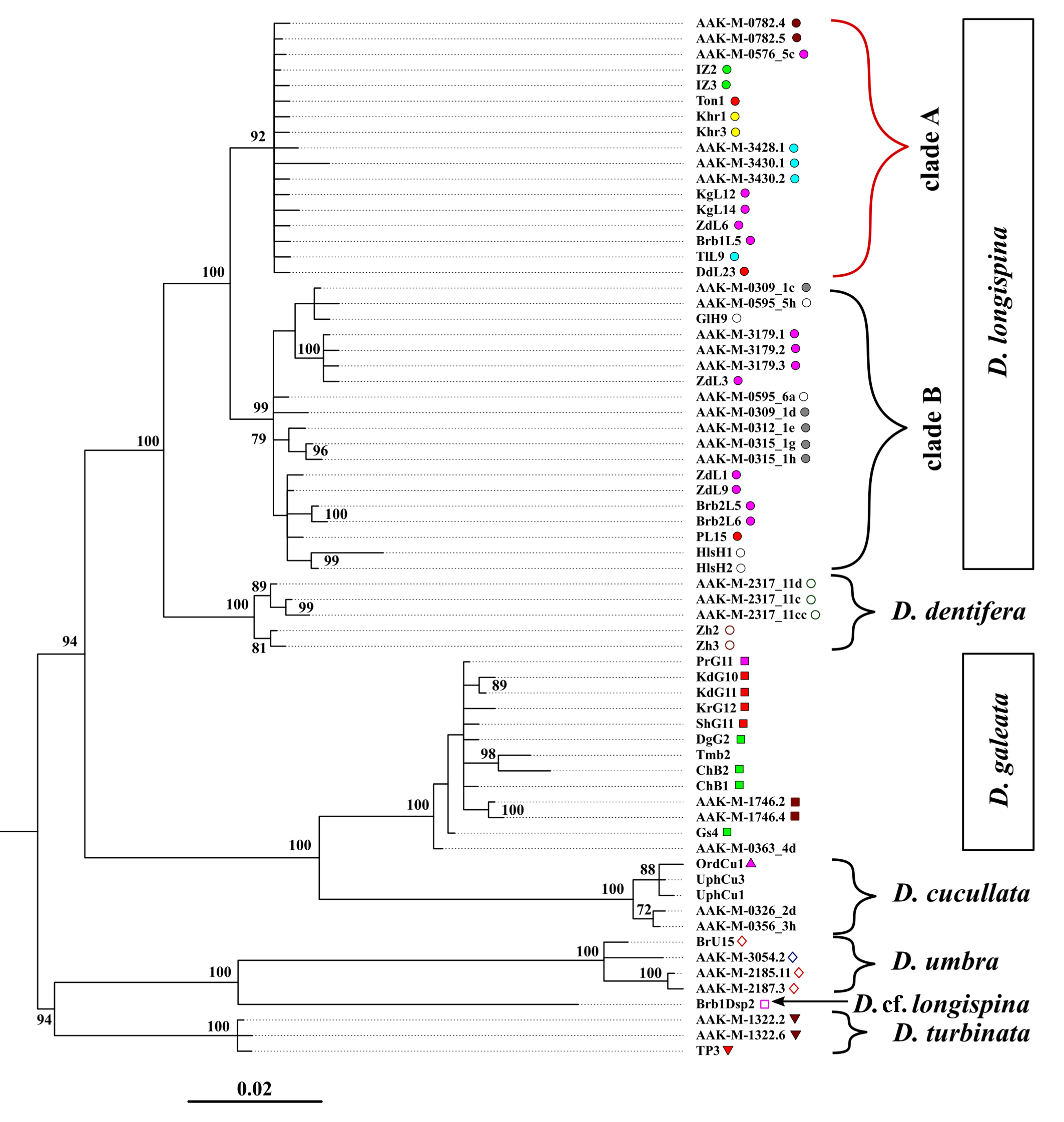

Supplement: S2 Fig — Bayesian posterior probabilities BI above 72% expressed as a percentage is indicated for each significant node. Scale is given in expected substitutions per site. Colored geometric symbols are the same as in S1 Fig. (TIF) [file pone.0221527.s009.tif]
